# Separation of Binding Protein of Celangulin V from the Midgut of *Mythimna separata* Walker by Affinity Chromatography

**DOI:** 10.3390/toxins7051738

**Published:** 2015-05-19

**Authors:** Lina Lu, Zhijun Qi, Jiwen Zhang, Wenjun Wu

**Affiliations:** 1 Institute of Pesticide Science, College of Plant Protection, Northwest A & F University, Yangling 712100, Shaanxi, China; E-Mails: linalusailboat@163.com (L.L.); qzhij@nwsuaf.edu.cn (Z.Q.); 2 College of Science, Northwest A & F University, Yangling 712100, Shaanxi, China; E-Mail: nwzjw@163.com

**Keywords:** Celangulin V, binding protein, affinity chromatography, *Mythimna separata* Walker

## Abstract

Celangulin V, an insecticidal compound isolated from the root bark of Chinese bittersweet, can affect the digestive system of insects. However, the mechanism of how Celangulin V induces a series of symptoms is still unknown. In this study, affinity chromatography was conducted through coupling of Celangulin V-6-aminoacetic acid ester to the CNBr-activated Sepharose 4B. SDS-PAGE was used to analyze the collected fraction eluted by Celangulin V. Eight binding proteins (Zinc finger protein, Thioredoxin peroxidase (TPx), Glyceraldehyde 3-phosphate dehydrogenase (GAPDH), SUMO E3 ligase RanBP2, Transmembrane protein 1, Actin, APN and V-ATPase) were obtained and identified by LC/Q-TOF-MS from the midgut of *Mythimna separata* larvae. The potential of these proteins to serve as target proteins involved in the insecticidal activity of Celangulin V is discussed.

## 1. Introduction

The disadvantages of synthetic pesticides, such as acute and chronic poisoning in humans, destruction of non-target organisms, environment contamination, and the evolution of resistance to pesticides in pest populations, have become more evident because of their increasing use [1]. By contrast, botanical pesticides have been receiving considerable attention because of their desirable properties of effectiveness, safety and ecological acceptability [2]. In addition to the outstanding and well-known bioactive compounds (pyrethrum, rotenone and nicotine), studies on bioactive compounds from other plants are increasing, such as species of the Annonaceae [3], the Meliaceae [4,5], the Celastraceae [6,7] and the Rutaceae [8,9], have been widely available. Moreover, the number of papers published annually on botanical pesticides increased from 61 in 1980 to 1207 in 2012 [10].

Chinese bittersweet (*Celastrus angulatus* Max), which belongs to the family Celastraceae, has long been known for its medicinal and insecticidal properties. The insecticidal potential of Celangulin derivates and various phytochemicals is isolated from the leaves, fruits and bark of the plant [11]. Wu *et al*. isolated and characterized a series of sesquiterpene polyesters sharing a dihydro-*b*-agarofuran sesquiterpenoid skeleton. Among these sesquiterpene polyesters, 44 possess insecticidal activity against several agricultural pests [12,13].

**Figure 1:**
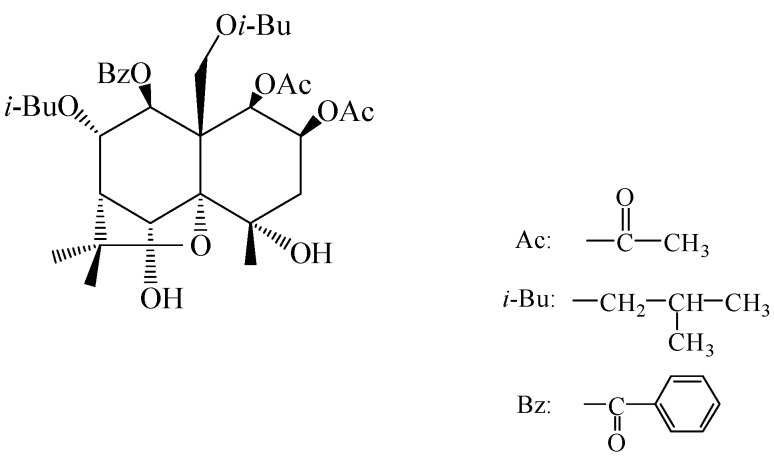
Structure of Celangulin V.

Celangulin V (Figure 1) is one of the insecticidal components isolated from the root bark of Chinese bittersweet [14]. It demonstrates insecticidal activity by causing pests to show a series of symptoms, such as excitement, twitching and loss of body fluid after oral administration [15,16]. Based on these symptomatological and anatomical studies, Wu *et al*. proposed the hypothesis that the Celangulin V has an effect on the digestive system of insects. Similar to Bt toxin, Celangulin V also causes the death of pests by affecting their midgut structure [17]. Lepidoptera insects are the most sensitive to Celangulin V [18]. Studies observed epithelial cells in the midgut of the Celangulin V-treated *Mythimna separata* Walker larvae under transmission electronmicroscope (TEM) showed that Celangulin V could induce time-dependent cytotoxicities in the midgut epithelial cells, such as visible vacuolization of cytoplasm, serious disruption of microvilli, fragmentation of RER cisternae, and rupture of plasma membrane. These morphological changes induce the leakage of cytoplasm contents into the midgut lumen and appearance of numerous lysosome-like vacuoles and secretion [16]. However, the insecticidal mechanism of Celangulin V is still not well understood. In this study, we separated the binding protein of Celangulin V from the midgut of *M. separata* larvae through affinity chromatography to better elucidate the mode of action of Celangulin V.

## 2. Results and Discussion

### 2.1. Identification of the Ligand (Celangulin V-6-aminoacetic Acid Ester)

**Scheme 1:**
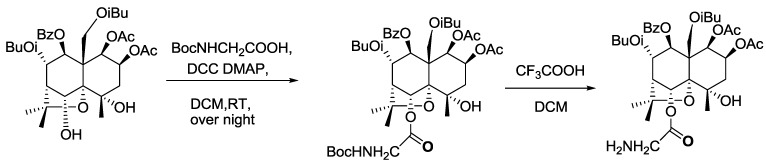
Synthetic route of ligand. *N,N*-dicyclohexylcarbodiimide (DCC), 4-dimethylaminopyridine (DMAP) and dichloromethane (DCM).

The ligand, Celangulin V-6-aminoacetic acid ester, can be synthesized via two reactions (Scheme 1) [19]. Compunds identifited by LC-MS and NMR are shown in the two following paragraphs. Later, the insecticidal activity of the ligand was determined through oral administration by feeding the fifth instar larvae of *M. separata.* The LD_50_ of ligand was 1.33 μg/mg (1.13-fold of Celangulin V), which indicates that t-butyloxycarboryl (Boc)-protected aminoacetic acid does not affect the insecticidal activity of the ligand. In other words, the 6-hydroxyl group could not be the active group, which is consistent with the result of our previous study on the structure-function relationship of compounds separated from Chinese bittersweet.

Celangulin V-6-Boc aminoacetic acid ester, C_41_H_57_NO_16_, an amorphous white powder, m.p. 86–88 °C. ESI-MS(*m/z*): 820 [M+H]^+^, 842 [M+Na]^+^. ^1^H-NMR (500 MHz, CDCl_3_) δ5.48 (1H, d, 3.5), 5.36 (1H, m), 2.10 (1H, m), 1.96 (1H, m), 6.55 (1H, s), 2.52 (1H, d, 2.5), 5.77 (dd, 1H, *J* = 9.5, 2.5 Hz), 6.03 (d, 1H, *J* = 9.5 Hz), 4.88, 4.65 (ABq, *J* = 13.0 Hz), 1.54 (s, 3H), 1.72 (s, 3H), 1.49 (s, 3H), 2.09 (s,3H), 1.56 (s, 3H), 2.94 (1H, m), 2.37 (1H, m), 1.41 (d, 3H, 7.0), 1.38 (d, 3H, 7.0), 0.92 (d, 3H, 7.0), 0.95 (d, 3H, 7.0), 7.86 (m, 2H), 7.56 (m, 1H), 7.41 (m, 2H). 6-Boc amino acetoxyl group: 4.00 (m, 2H), 1.45 (s, 9H). ^13^C-NMR (125 MHz, CDCl_3_) δ75.1 (CH), 67.6 (CH), 42.2 (CH_2_), 69.8 (C), 92.1 (C), 76.2 (CH), 52.2 (CH), 73.3 (CH), 75.4 (CH), 51.4 (C), 84.4 (C), 61.7 (CH_2_), 24.8 (CH_3_), 25.9 (CH_3_), 29.8 (CH_3_), 169.6 (CO), 169.5 (CO), 21.1 (CH_3_), 20.5 (CH_3_), 176.9 (CO), 175.6 (CO), 34.3 (CH), 34.1 (CH), 19.1 (CH_3_), 18.9 (CH_3_), 18.6 (CH_3_), 18.5 (CH_3_), 165.6 (CO), 133.5 (CH), 129.5 (CH × 2), 129.3 (C), 128.7 (CH × 2). 6-Boc amino acetoxyl group: 43.1 (CH_2_), 168.8 (CO), 155.6 (CO), 80.1 (C), 28.3 (CH_3_ × 3).

Celangulin V-6-aminoacetic acid ester, C_36_H_49_NO_14_, an amorphous white powder, m.p. 122–124 °C. ESI-MS(*m/z*): 720 [M+H]^+^, 742 [M+Na]^+^. ^1^H-NMR (500 MHz, CDCl_3_) δ5.45 (1H, d, 3.5), 5.30 (1H, m), 2.19 (1H, m), 1.95 (1H, m), 6.62 (1H, s), 2.74 (1H, d, 2.5), 5.83 (dd, 1H, *J* = 9.5, 2.5 Hz), 6.08 (d, 1H, *J* = 9.5 Hz), 4.96, 4.69 (ABq, *J* = 13.0 Hz), 1.54 (s, 3H), 1.72 (s, 3H), 1.51 (s, 3H), 2.09 (s, 3H), 1.56 (s, 3H), 2.92 (1H, m), 2.38 (1H, m), 1.40 (d, 3H, 7.0), 1.35 (d, 3H, 7.0), 0.92 (d, 3H, 7.0), 0.88 (d, 3H, 7.0), 7.92 (m, 2H), 7.66 (m, 1H), 7.52 (m, 2H). 6-amino acetoxyl group: 3.26 (m, 2H). ^13^C-NMR (125 MHz, CDCl_3_) δ76.2 (CH), 68.3 (CH), 42.3 (CH_2_), 70.5 (C), 92.9 (C), 78.4 (CH), 52.8 (CH), 74.1 (CH), 76.6 (CH), 52.5 (C), 85.2 (C), 62.4 (CH_2_), 25.6 (CH_3_), 26.1 (CH_3_), 29.8 (CH_3_), 169.9 (CO), 169.8 (CO), 20.9 (CH_3_), 20.7 (CH_3_), 176.7 (CO), 175.8 (CO), 35.0 (CH), 34.7 (CH), 19.5 (CH_3_), 19.4 (CH_3_), 18.9 (CH_3_), 18.8 (CH_3_), 165.5 (CO), 134.5 (CH), 130.3 (CH × 2), 130.5 (C), 129.6 (CH × 2). 6-amino acetoxyl group: 43.2 (CH_2_), 166.4 (CO).

### 2.2. Separation of Binding Protein

**Figure 2:**
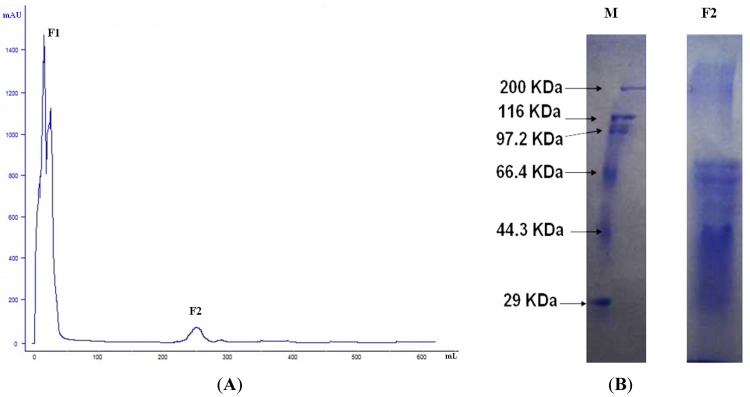
Affinity chromatography using Celangulin V-Sepharose of BBMV proteins extracts from *M. separata* larvae. (**A**) F1 is the unbound protein eluted with binding buffer; F2 is eluted with Celangulin V dissolved in binding buffer; (**B**) 12% SDS-PAGE of fraction M: protein marker; F2: Fraction 2.

Epithelial cells are the first cells to come into contact with Celangulin V after its entrance to the midgut. Earlier research has shown that Celangulin V can destroy microvilli and the organelles of midgut epithelial cells, thus, we speculated that the existence of the binding protein of Celangulin V on the midgut cell membranes is plausible. We separated the binding protein by using affinity chromatography after isolating the brush border membrane vesicle (BBMV) of *M. separata* larvae. Two fractions, F1 and F2, were present. F1 is the unbound proteins eluted with binding buffer, whereas F2 was eluted with Celangulin V dissolved in binding buffer (Figure 2A).

SDS-PAGE (Figure 2B) and LC/Q-TOF-MS were conducted to resolve and analyze, respectively, the proteins eluted by dissociative Celangulin V. Table 1 shows the binding proteins by searching the insecta database.

**Table 1:** Midgut proteins from *M. separata* recognized by affinity chromatography.

Number	Protein	Genbank gi number	Species
1	Actin	gi|108879764	*Aedes aegypti*
2	Zinc finger protein	gi|157127505	*Aedes aegypti*
3	Thioredoxin peroxidase	gi|7230426	*Drosophila melanogaster*
4	Glyceraldehyde-3-phosphate dehydrogenase	gi|112983816	*Bombyx mori*
5	Transmembrane protein 1	gi|108872951	*Aedes aegypti*
6	E3 SUMO-protein ligase RanBP2	gi|307201149	*Harpegnathos saltator*
7	Amino peptidase N3	gi|21327773	*Plutella xylostella*
8	Protease m1 zinc metalloprotease	gi|108875833	*Aedes aegypti*
9	Vacuolar ATPase subunit a	gi|5852166	*Manduca sexta*
10	Vacuolar ATPase subunit B	gi|8810	*Drosophila melanogaster*
11	Vacuolar ATPase subunit H	gi|5852164	*Manduca sexta*

Among the binding proteins obtained from affinity chromatography, zinc finger protein, thioredoxin peroxidase (TPx), glyceraldehyde 3-phosphate dehydrogenase (GAPDH) and SUMO E3 ligase RanBP2 were mainly located in the cytoplasm but not on the plasma membrane. These proteins have diverse functions, such as regulating gene expression, protecting cells from apoptosis, participating in metabolic pathways and so on [20,21,22,23,24,25,26]. However, the symptoms induced by malfunction of these proteins are not relevant to the symptoms caused by Celangulin V. Hence, those proteins are irrelevant target proteins.

Our previous study showed that the larvae lost body fluid and the midgut epithelial cell microvilli were seriously disrupted. Given this finding, we can deduce that Celangulin V may destroy the cell microvilli by interacting with proteins that exist on the membrane or proteins which associate with microvillar membrane to affect their normal functions. In addition, because the symptoms caused by Celangulin V are similar to those of Bt toxin, Celangulin V may have the same target protein as Bt toxin. Therefore, the possible putative target proteins could be APN3 and V-ATPase. Actin is reported to increase in larval midgut after Cry1Ac toxin ingestion. Because actin supports the apical surface of the brush border in the midgut, the increases of actin could provide protection to enhancing cellular defenses [27]. In addition to APN and V-ATPase, we also found actin when the affinity chromatography was conducted. Actin is one of the high abundance proteins in all eukaryotic cells; it forms microfilaments and has multi-functions, such as cell motility, cell division, organelle movement, and the maintenance of cell junctions and cell shape. Many of these processes are mediated by extensive and intimate interactions of actin and cellular membranes [28]. In consideration of the selectivity, actin has less possibility to be target protein of drugs and pesticides because it is highly conservative and abundant in cell.

Aminopeptidase N (APN) exists in plants and animals and is an aminopeptidase that hydrolyzes N-terminal neutral amino acid of protein or peptides. In insects, APN is mainly located at the brush border membrane of the midgut and is involved in the digestion of protein in food. APN has been extensively studied as one of the receptors of insecticidal crystal proteins of *Bacillus thuringiensis*. Insect APN has five classes (class 1 to 5). Among these five classes, APN3 is similar to APN1, which is rich in threonine at the C-terminal. However, APN3 has less O-glycosylation sites [29]. APN3 of *Manduca sexta* is expressed in *Trichoplusiani ni* cell line. Furthermore, the usage of a specific chemical deglycosylation technique demonstrated that carbohydrates attached to the 120 kDa APN3 are the binding epitopes for Cry1Ac toxin instead of Cry1Ab or Cry1Aa [30]. Tetreau *et al.* [31] mentioned the protease m1 zinc metalloprotease as N-aminopeptidase protein in *Aedes aegypti* about the *Bti* resistance. Celangulin V can affect the digestive system like Bt toxin, but in contrast to Bt toxin, Celangulin V is a small molecule, which cannot insert into the cell membranes to create ion channels or pores [32]. Moreover, our APN activity assay data showed that APN activity was not affected by Celangulin V the *in vitro* (data not shown). In that way, APN can also be excluded from the putative target proteins of Celangulin V.

Therefore, V-ATPase is the most likely putative target protein. V-ATPase, exists in endomembranes and plasma and is one of the fundamental enzymes in organisms [33]. V-ATPase plays a critical role in acidifying specific organelles in endomembranes and participates in pH homeostasis and membrane energization in plasma membranes. Insect V-ATPase is involved in transepithelial cation transport in epithelia by cooperating with K^+^/H^+^ antiporter and ion channels. For Lepidoptera larvae, V-ATPase is responsible for the alkalinization of gut lumen [34,35]. Given the important function of V-ATPase, it can be used as a drug target. Many inhibitors of V-ATPase have been reported and studied extensively [36]. Insect V-ATPase is also reported to be possibly one of the receptors of Bt toxin, and its expression level in *Bti* resistant strain of yellow fever mosquitoes altered [31]. A significant increase was observed in V-ATPase subunits in BBMV of *Helicoverpa armigera* after ingesting Cry1Ac toxin [27].

In conclusion, these eight proteins obtained from affinity chromatography are potential binding proteins interacting with Celangulin V, and V-ATPase might be the target protein of Celangulin V to induce a series of symptoms to destroy the digestive system. However, further studies are necessary to confirm and support this conclusion.

## 3. Experimental Section

### 3.1. Insects

Laboratory-adapted *M. separata* (Walker) was obtained from the Institute of Pesticide Science, Northwest A & F University (NWAFU). The strain was reared on wheat and corn leaves under laboratory conditions for about 20 years, and was never in contact with insecticides.

### 3.2. Chemicals

Celangulin V (Purity > 98% according to HPLC analysis) was provided by the Institute of Pesticide Science, NWAFU. CNBr-activated Sepharose 4B was purchased from GE Healthcare (Beijing, China). Tris, NaCl, HCl, acetic acid, sodium acetate and all other chemicals were purchased from AMRESCO (Solon, OH, USA) and Guanghua Sci-Tech (Guangzhou, China). DMF (*N,N*-dimethylformamide) was purchased from TEDIA (Shanghai, China).

### 3.3. Isolation of BBMV from M. Separata Walker

Sixth instar larvae of *M. separata* Walker were starved for 12 h. Midguts were then removed from the larvae and peritrophic membrane and gut contents were discarded. The midguts were washed in ice-cold 0.7% NaCl solution, and the cleaned tissue was weighed and used to isolate the BBMV according to the MgCl_2_ precipitation method [37], as modified by Ferre *et al*. [38]. the final pellet was dissolved in buffer C (150 mM NaCl, 5 mM EGTA, 1 mM PMSF, 20 mM Tris-HCl, 1% CHAPS) [39]. The protein concentration of BBMV was measured by Bradford Assay.

### 3.4. Synthesis of Ligand

The ligand, Celangulin V-6-aminoacetic acid ester, was synthesized using Celanglin V and t-butyloxycarboryl (Boc)-protected aminoacetic acid (Scheme 1).

#### 3.4.1. Synthesis of Celangulin V-6-Boc Aminoacetic Acid Ester

One hundred milligrams of Celangulin-V (0.15 mmol) was dissolved in 10 mL of anhydrous methylene chloride, and Boc-protected amino acetic acid (32 mg, 0.18 mmol), *N,N'*-dicyclohexylcarbodiimide (DCC, 62 mg, 0.30 mmol), and 4-dimethylaminopyridine (DMAP) (5 mg, 0.04 mmol) were then added. The mixture was stirred over night at room temperature. When the reaction was completed (checked by TLC), 1 mL of methanol was added to quench the reaction. After filtration, 40 mL of water was added to the mixture which was then extracted with ethyl acetate (30 mL × 3). Ethyl acetate layers were combined and washed with water and saturated sodium chloride, dried over anhydrous sodium sulfate and separated by column chromatography(silica gel, 200~300 mesh) with a gradient of petroleum ether (60–90 °C) and ethyl acetate as eluent to yield a compound of 76 mg of Celangulin V-6-Boc aminoacetic acid ester. The structure of the compound was characterized by ^1^H-NMR, ^13^C-NMR, DEPT and MS.

#### 3.4.2. Synthesis of Celangulin V-6-aminoacetic Acid Ester

Eighty-two milligrams (0.1 mmol) of Celangulin V-6-Boc aminoacetic acid ester obtained from the last step was dissolved in 10 mL of anhydrous methylene chloride. After adding trifluoroacetic acid (0.2 mL, 1.75 mmol), the mixture was stirred overnight at room temperature. The reaction was checked with TLC, and when it was completed, 1 mL of saturated sodium bicarbonate solution was added to quench the reaction. Twenty milliliters of water was added to the mixture which was then extracted with ethyl acetate (30 mL × 3). Ethyl acetate layers were combined and washed with water and saturated sodium chloride, dried over anhydrous sodium sulfate and separated by column chromatography (silica gel, 200 ~ 300 mesh) with a gradient of petroleum ether (60–90 °C) and ethyl acetate as eluent to yield a compound of 76 mg of Celangulin V-6-aminoacetic acid ester. The structure was characterized by ^1^H-NMR, ^13^C-NMR, DEPT and MS.

### 3.5. Preparing the Medium and Coupling the Ligand

The medium, CNBr-actived Sepharose 4B, was prepared according to the manual. Lyophilized powder (2.5 g) was weighed and suspended in 100 mL of 1 mM HCl. The medium swelled immediately and was washed for 15 min. Approximately 500 mL of HCl was used, and the medium was washed five times.

The ligand (Celangulin V-6-aminoacetic acid ester) was dissolved in coupling buffer, 0.1 M NaHCO_3_, pH 8.3 containing 0.5 M NaCl and 30% DMF. The coupling solution containing the ligand was mixed with the prepared medium, and the mixture was rotated overnight at 4 °C. The excess ligand was then washed away by using 5 gel volumes of coupling buffer. To block any remaining active groups, we transferred the medium coupled with ligand to 0.1 M Tris-HCl buffer, and incubated for 2 h at room temperature. Finally, the medium was washed with three cycles of alternating pH buffer. The volumes of each buffer were five times the medium volumes. For each cycle, the medium was washed with 0.1 M acetic acid/sodium acetate, pH 4.0 containing 0.5 M NaCl, and then with 0.1 M Tris-HCl, pH 8.0 containing 0.5 M NaCl.

### 3.6. Binding, Elution, and Regeneration

The prepared Sepharose 4B was packed to the column of AKTA protein purification system (GE Healthcare, Beijing, China). The packing flow rate was maintained for 3 bed volumes after a constant bed height was reached.

After 12 mL BBMV (3.02 mg/mL) of *M. separata* Walker was loaded, the medium was washed with the binding buffer (0.1 M NaH_2_PO_4_/Na_2_HPO_4_, pH 8.0 containing 0.5 M NaCl) until the base line is stable. For competitive elution, Celangulin V was dissolved in binding buffer. Elution peak was collected by washing the medium with elution buffer. To regenerate the medium, we washed the affinity medium with alternating high pH (0.1 M Tris-HCl, pH 8.0 containing 0.5 M NaCl) and low pH (0.1 M acetic acid/ sodium acetate, pH 4.0 containing 0.5 M NaCl) buffer. And this cycle was repeated three times.

### 3.7. SDS-PAGE and LC/Q-TOF-MS

Dialysis method was used to decrease the high salt concentration in the collected eluate. The sample was then lyophilized for the following procedure.

SDS-PAGE with 12% separating gel was carried out by dissolving the sample in the Tris-HCl (pH 7.2). Subsequently, the gel was cut for identification. Protein mass spectrometry was performed in Beijing Protein Institute by using LC/Q-TOF-MS. The gel was digested by trypsin at 37 °C overnight, and after terminating the reaction, 10 μL of sample was analyzed by mass spectrometry.

## 4. Conclusions

Eight potential binding proteins of Celangulin V were separated from the midgut of *M. separata* larvae through affinity chromatography, to provide the basis for the mode of action of Celangulin V. These potential binding proteins include zinc finger protein, TPx, GAPDH, SUMO E3 ligase RanBP2, transmembrane protein 1, actin, APN and V-ATPase. The analysis of the functions of these binding proteins, as well as the symptoms induced by Celangulin V, we speculated that the putative target protein could be V-ATPase. Further studies are still required to confirm this hypothesis.

## References

[1] Isman M.B. (2006). Botanical insecticides, deterrents, and repellents in modern agriculture and an increasingly regulated world. Annu. Rev. Entomol..

[2] Rosell G., Quero C., Coll J., Guerrero A. (2008). Biorational insecticides in pest management. J. Pestic. Sci..

[3] Grzybowski A., Tiboni M., Silva M.A., Chitolina R.F., Passos M., Fontana J.D. (2013). Synergistic larvicidal effect and morphological alterations induced by ethanolic extracts of Annona muricata and Piper nigrum against the dengue fever vector Aedes aegypti. Pest Manag. Sci..

[4] He L., Wang L. (2012). A brief review on insecticidal function of azadirachta indica. Pestic. Sci. Adm..

[5] Wang L., He L., Shen Y. (2013). Advances in studies on insecticidal function of meliaceae. Pestic. Sci. Adm..

[6] Deepa M.A., Bai V.N. (2010). Bioinsecticidal compounds of celastraceae-the spindle tree family. Int. J. Bot..

[7] Wei S., Wang M., Ji Z., Shi B., Li S., Zhang J. (2010). Three new insecticidal sesquiterpene polyol esters from *Celastrus angulatus*. Nat. Prod. Commun..

[8] Karamaouna F., Kimbaris A., Michaelakis A., Papachristos D., Polissiou M., Papatsakona P., Tsora E. (2013). Insecticidal activity of plant essential oils against the vine mealybug, *Planococcus ficus*. J. Insect Sci..

[9] Raturi R., Badoni P.P., Ballabha R. (2014). Insecticidal and fungicidal activities of stem bark of *Zanthoxylum Armatum* (Rutaceae). Word J. Pharm. Pharm. Sci..

[10] Isman M.B., Grieneisen M.L. (2014). Botanical insecticide research: many publications, limited useful data. Trends Plant Sci..

[11] Gao J.M., Wu W.J., Zhang J.W., Konishi Y. (2007). The dihydro-β-agarofuran sesquiterpenoids. Nat. Prod. Rep..

[12] Ji Z., Wu W., Yang H., Shi B., Wang M. (2007). Four novel insecticidal sesquiterpene esters from *Celastrus angulatus*. Nat. Prod. Res..

[13] Ji Z., Zhang Q., Shi B., Wei S., Wang M., Wu W. (2009). Three new insecticidal sesquiterpene pyridine alkaloids from *Celastrus angulatus*. Nat. Prod. Res. Former. Nat. Prod. Lett..

[14] Wu W.J., Li S.B., Zhu J.B., Liu H.X. (1994). New sequiterpenoid CelangulinV: Isolation and determination. Acta Univ. Agric. Boreali-Occidentalis.

[15] Yang R.Y., Liu H.X., Wu W.J., Wang J.L. (2001). Study on the functioning mechanism of celangulin V. Northwest Sci.-Technol. Univ. Agri. For. (Nat. Sci. Ed.).

[16] Qi Z., Shi B., Hu Z., Zhang Y., Wu W. (2011). Ultrastructural effects of Celangulin V on midgut cells of the oriental armyworm, *Mythimna separata* walker (Lepidoptera: Noctuidae). Ecotoxicol. Environ. Saf..

[17] Wu W.J., Ji Z.Q., Hu Z.N. (1997). Natural products and digestive poisons. Pesticides.

[18] Wu W.J., Hu Z.N., Liu H.X., Qi Z.J. (2005). Insecticidal mechanisms of the major active components from the Chinese bittersweet, *Celastrus angulatus* and their application. Acta Entomol. Sin..

[19] Zhang J., Hu Z., Li S., Wu W. (2011). Synthesis and insecticidal activities of new ester-derivatives of Celangulin-V. Int. J. Mol. Sci..

[20] Zhao N., Zhao F., Li Y. (2009). Advances in research on zinc finger protein. Lett. Biotechnol..

[21] Zhang P., Liu B., Kang S.W., Seo M.S., Rhee S.G., Obeid L.M. (1997). Thioredoxin peroxidase is a novel inhibitor of apoptosis with a mechanism distinct from that of Bcl-2. J. Biol. Chem..

[22] Tarze A., Deniaud A., Bras M.L., Maillier E., Molle D., Larochette N., Zamzami N., Jan G., Kroemer G., Brenner C. (2006). GAPDH, a novel regulator of the pro-apoptotic mitochondrial membrane permeabilization. Oncogene.

[23] Zala D., Hinckelmann M.-V., Yu H., Cunha M.M.L.D., Liot G., Cordelières F.P., Marco S., Saudou F. (2013). Vesicular glycolysis provides on-board energy for fast axonal transport. Cell.

[24] Hay R.T. (2005). SUMO: A history of modification. Mol. Cell.

[25] Pichler A., Gast A., Seeler J.S., Dejean A., Melchior F. (2002). The nucleoporin RanBP2 has SUMO1 E3 ligase activity. Cell.

[26] Kirsh O., Seeler J.S., Pichler A., Gast A., Muller S., Miska E., Mathieu M., Harel-Bellan A., Kouzarides T., Melchior F. (2002). The SUMO E3 ligase RanBP2 promotes modification of the HDAC4 deacetylase. EMBO J..

[27] Yuan C., Ding X., Xia L., Yin J., Huang S., Huang F. (2011). Proteomic analysisi of BBMV in *Helicoverpa armigera* midgut with and without Cry1Ac toxin treatment. Biocontrol Sci. Technol..

[28] Doherty G.J., McMahon H.T. (2008). Mediation, modulation, and consequences of membrane-cytoskeleton interactions. Annu. Rev. Biophys..

[29] Chang X., Wu Q., Wang S., Xu B., Zhang Y. (2011). Studying progress on aminopeptidase N of insects. Chin. J. Pestic. Sci..

[30] Knight P., Carroll J., Ellar D. (2004). Analysis of glycan structures on the 120 kDa aminopeptidase N of *Manduca sexta* and their interactions with *Bacillus thuringiensis* Cry1Ac toxin. Insect Biochem. Mol. Biol..

[31] Tetreau G., Bayyareddy K., Jones C.M., Stalinski R., Riaz M.A., Paris M., David J.-P., Adang M.J., Després L. (2012). Larval midgut modifications associated with Bti resistance in the yellow fever mosquito using proteomic and transcriptomic approaches. BMC Genomics.

[32] Schnepf E., Crickmore N., Rie J.V., Lereclus D., Baum J., Feitelson J., Zeigler D.R., Dean D.H. (1998). *Bacillus thuringiensis* and its pesticidal crystal proteins. Microbiol. Mol. Biol. Rev..

[33] Nelson N. (2003). A journey from mammals to yeast with vacuolar H^+^-ATPase (V-ATPase). J. Bioenerg. Biomembr..

[34] Wieczorek H., Huss M., Merzendorfer H., Reineke S., Vitavska O., Zeiske W. (2003). The insect plasma membrane H^+^ V-ATPase: Intra-, Inter-, and supramolecular aspects. J. Bioenerg. Biomembr..

[35] Wieczorek H., Beyenbach K.W., Huss M., Vitavska O. (2009). Vacuolar-type proton pumps in insect epithelia. J. Exp. Biol..

[36] Bowman E.J., Bowman B.J. (2005). V-ATPases as drug targets. J. Bioenerg. Biomembr..

[37] Wolfersberger M.G., Luthy P., Maurer A., Parenti P., Sacchi V.F., Giordana B., Hanozet G.M. (1987). Preparation and partial characterization of amino acid transporting brush border membrane vesicles from the larval midgut of the cabbage butterfly (*Pieris brassicae*). Comp. Biochem. Physiol. Part A Physiol..

[38] Ferré J., Real M.D., Rie J.V., Jansens S., Peferoen M. (1991). Resistance to the *Bacillus thuringiensis* bioinsecticide in a field population of *Plutella xylostella* is due to a change in a midgut membrane receptor. Proc. Natl. Acad. Sci. USA.

[39] Chen L.Z., Liang G.M., Zhang J., Wu K.M., Guo Y.Y., Rector B.G. (2010). Proteomic analysis of novel Cry1Ac binding proteins in Helicoverpa armigera (Hübner). Arch. Insect Biochem. Physiol..

